# The Potential of a Novel Cold Atmospheric Plasma Jet as a Feasible Therapeutic Strategy for Gingivitis—A Cell-Based Study

**DOI:** 10.3390/cells13231970

**Published:** 2024-11-28

**Authors:** Andreea-Mariana Negrescu, Leonardo Zampieri, Emilio Martines, Anisoara Cimpean

**Affiliations:** 1 Department of Biochemistry and Molecular Biology, Faculty of Biology, University of Bucharest, 91-95 Spl. Independentei, 050657 Bucharest, Romania; andreea-mariana.negrescu@bio.unibuc.ro; 2 Research Institute of the University of Bucharest (ICUB), University of Bucharest, 050657 Bucharest, Romania; 3 Department of Physics “Giuseppe Occhialini”, University of Milano-Bicocca, Piazza Della Scienza 3, 20126 Milan, Italy; l.zampieri4@campus.unimib.it (L.Z.); emilio.martines@unimib.it (E.M.)

**Keywords:** cold atmospheric plasma, gingivitis, gingival fibroblasts, macrophages, wound healing, inflammation

## Abstract

Due to its antimicrobial, anti-inflammatory and pro-healing properties, the application of cold atmospheric plasma (CAP) has emerged as a new and promising therapeutic strategy in various fields of medicine, including general medicine and dentistry. In this light, the aim of the present study was to investigate the effects of a homemade plasma jet on the cellular behaviour of two important cell types involved in gingivitis, namely gingival fibroblasts (HGF-1 cell line) and macrophages (RAW 264.7 cell line), by the direct application of CAP in different experimental conditions. The cellular behaviour of the HGF-1 cells was investigated in terms of viability/proliferation (LIVE/DEAD and CCK-8 assays), morphological features (immunofluorescent staining of the actin cytoskeleton) and fibronectin expression (immunocytochemical staining of the fibronectin network), while the macrophages’ response was evaluated through the assessment of the cellular survival/proliferation rate (LIVE/DEAD and CCK-8 assays), morphological behaviour (immunofluorescent staining of the actin cytoskeleton) and inflammatory activity (pro-inflammatory cytokine secretion profile (ELISA assay) and foreign body giant cells (FBGCs) formation (immunofluorescent staining of the actin cytoskeleton and multinuclearity index determination)). The in vitro biological assessment revealed an upward trend dependent on treatment time and number of CAP applications, in terms of fibroblasts proliferation (*p* < 0.0001) and fibronectin expression (*p* < 0.0001). On the other hand, the macrophages exposed to five consecutive CAP applications for longer treatment times (over 120 s) exhibited a strong pro-inflammatory activity, as evinced by their altered morphology, pro-inflammatory cytokine profile (*p* < 0.0001) and FBGCs formation. Overall, our results demonstrate that CAP exposure, when used with appropriate operating parameters, has a beneficial effect on the cellular response of HGF-1 and RAW 264.7 cells, thus paving the way for further in vitro and in vivo investigations that will allow the translation of CAP treatment from research to clinic as an alternative therapy for gingivitis.

## 1. Introduction

Due to its high prevalence and global incidence, as well as its impact on the individuals’ quality of life and its association with various systemic conditions, periodontal disease has been recognised by the World Health Organization (WHO) as a significant public health concern, highlighting the urgency of periodontal care and new treatment strategies [1,2,3,4]. Influenced by a multitude of factors such as host conditions (e.g., innate and adaptive immune responses) and external factors (e.g., dental plaque accumulation, smoking, stress, the socio-economic background, etc.), periodontal disease is a microbial-associated, host-mediated chronic inflammatory state, characterised by the progressive destruction of the soft (gingiva and periodontal ligaments) and hard (alveolar bone and cementum) tissues adjacent to the teeth [5].

Amongst all of the periodontal conditions, gingivitis is the most prevalent form, with a higher incidence in males as opposed to females and in individuals belonging to lower socio-economic groups [6,7,8,9]. As opposed to periodontitis, gingivitis is a localised condition, which affects the gingival epithelium and the underlying connective tissue, being clinically characterised by tissue redness, tenderness, swelling and bleeding upon gentle probing [10]. Based on its aetiology, clinical presentation and duration of infection, various forms of gingivitis have been identified (e.g., plaque-, drug-induced gingivitis, nutritional and hormonal gingivitis), with the plaque-induced condition being one of the most frequently observed variants [6]. As reflected by its name, the plaque-induced gingivitis has a pathogenic aetiology, in which the continuous accumulation of oral biofilm without removal or disruption, in or in close proximity to the gingival sulcus, results in injury to the periodontium through direct and indirect mechanisms that involve the activation and propagation of the host tissue inflammatory responses [11]. From a histopathological point of view, the gingiva undergoes a series of alterations such as the elongation of the rete ridges into the gingival connective tissue, the dilatation of the blood vessels bordering the junctional epithelium, the graduate destruction of the collagen fibres with changes in the synthetized collagen type, morphological modifications in the resident fibroblasts and a progressive inflammatory infiltrate [12,13].

To date, the traditional therapy scheme for individuals with gingivitis is focused on reducing the oral bacterial load, either through a self-care oral hygiene regime or the professional management of the dental biofilm [14]. The oral hygiene regime maintained at home consists in an improved dental cleaning routine, which includes a regular and appropriate tooth brushing technique coupled with the practice of interproximal hygiene [15,16,17]. However, even though data reported in the literature demonstrates the feasibility of plaque control only through an improvement in the personal oral hygiene routine, the epidemiological reports suggest that the majority of the affected individuals are missing either the requisite motivation or the competence to adhere and sustain a plaque-free status [18,19,20]. Moreover, various clinical trials have demonstrated that, despite the meticulous self-administered plaque control regime, some amount of dental biofilm is still left behind in undetected areas [15,21,22]. For example, various dental anatomical structures such as deep groves, cervical enamel projections, furcation and concavities can create spaces in which the dental plaque remains hidden and out of reach of the toothbrush [23,24]. Through scaling, the professional removal of dental plaque and calculus is obtained, even from those inaccessible areas [14]. However, the professional management of biofilm has its own disadvantages and does not represent a long-term solution, mainly due to its inability to completely eliminate the periodontal pathogens, leading in time to a recolonisation to pre-treatment levels within weeks and to the reappearance of a more pathogenic plaque within months [10]. Furthermore, this conventional treatment strategy is uncomfortable for patients and is technically demanding [15]. Apart from the mechanical removal of plaque, the use of topical antibacterial agents may be prescribed in the prevention and treatment of gingivitis, but as the experimental evidence indicates, the penetration of the topically applied antibacterial agents into the gingival sulcus is quite challenging and minimal [15,25,26].

In this light, the need for new adjuvant therapies for gingivitis that not only are capable of reducing the pathogenic dental plaque but also modulate the host immune response and promote tissue healing has increased in recent years, the reason why the scientific community has turned its attention to a relatively newcomer in medicine, namely plasma technology. Low-temperature plasmas, also known as cold atmospheric plasmas (CAPs), are generated when the kinetic energy of the electrons enclosed by plasma is higher than that of the ions and molecules found in the neutral gas, leading to a discharge of a non-equilibrium plasma when an electric field is applied [27,28]. Due to ability of these low-temperature plasmas (CAPs) to operate at or near room temperature, they can be safely applied to various biological substrates without causing any thermal damages [28]. In addition to being biologically safe, CAPs also possess several biological active components, for example, reactive oxygen (ROS) and nitrogen (RNS) species, ions and electrons, which, through their synergistic action, can elicit a variety of biological responses within various forms of plasma treatment [29,30]. Furthermore, the small dimensions of the plasma source facilitate its use in the buccal cavity, while the plasma jet can be applied with ease to the whole oral cavity’s non-uniform surface [31]. Moreover, an additional benefit of CAP is the relatively low manufacturing cost of the plasma-generating source, which can help reduce the financial burden imposed by the conventional therapies on the healthcare system [32]. Nevertheless, as stated previously, CAP generation is accompanied by an electric field and photon emission; therefore, in order for CAP to be used safely to treat the alterations of various cell types and biological tissues, a suitable source and operating parameters for plasma generation should be carefully selected to avoid any thermal damages but maintain, at the same time, the plasma’s efficacy [30].

Even though, in the last few years, the CAP treatment has been applied with success in biomedicine [27,33], mainly in dermatology (e.g., disinfection [34], wrinkle smoothing [35,36], atopic dermatitis [37,38], wound healing [39,40], scar treatment [41], etc.), oncology [42,43,44,45,46,47,48], sterilisation, infection control [49] and even dentistry (e.g., dental implant sterilisation and tooth whitening) [33,50,51,52], studies that focus on the potential of CAP as a feasible therapeutic strategy for various pathological conditions associated with oral infections are very scarce, despite the technique’s great potential. On this basis, the present study seeks to ascertain the efficacy of cold atmospheric plasma as a possible therapeutic strategy for gingivitis. To this end, the effects of a homemade plasma source on two cellular types involved in the pathogenesis of gingivitis, namely the gingival fibroblasts and macrophages, were investigated.

## 2. Materials and Methods

### 2.1. Cold Atmospheric Plasma Jet

**Figure 1:**
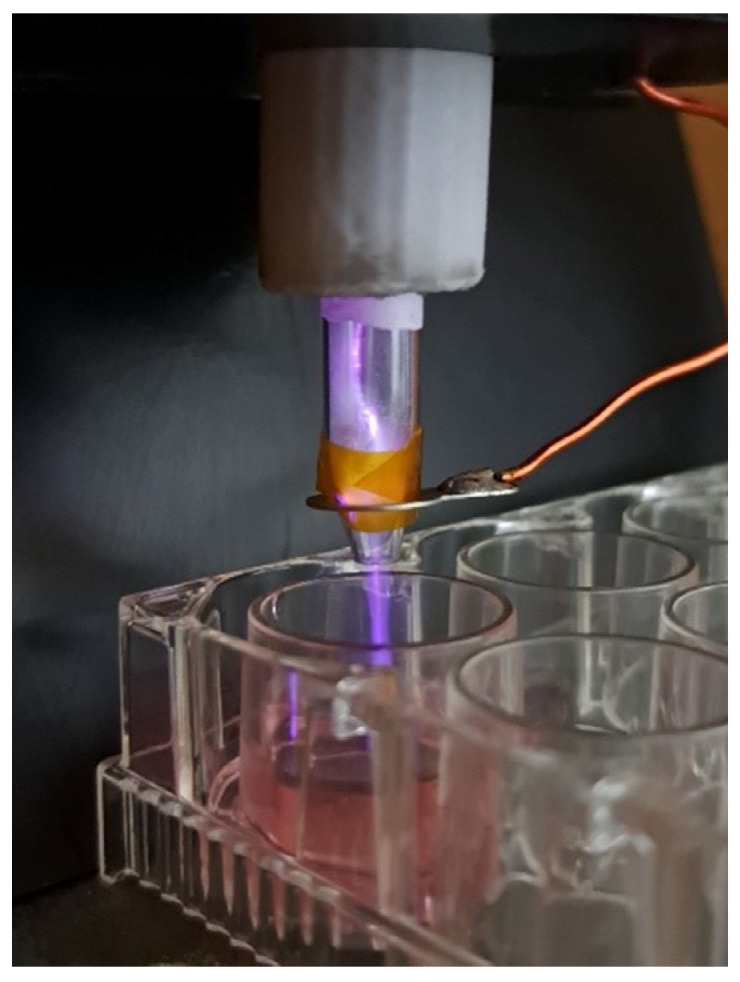
CAP source during cell culture treatment.

PlasmaBIC (Figure 1) is a CAP source designed and produced in the laboratories of the University of Milan-Bicocca, developed to be simple and cheap to reproduce, compact and hand-held. The source layout is composed of an axially symmetric double-dielectric jet: a central copper rod covered by a glass capillary (external diameter 1.4 mm) and enclosed in a gas nozzle (internal diameter 6 mm). A ground ring, outside from the nozzle, completes the setup. The central electrode is connected to a transformer, placed on the source itself (1:100 ratio) and the primary winding is upper-hanging and pulled to the ground with a squared wave. The frequency of the wave has been fixed at 100 kHz to match the resonant frequency of the transformer, while the pulse width can be varied to cover the delivered power and, in this work, has been fixed at 50%. A 1 kHz 20% duty cycle was superimposed to the power supply to prevent the transformer from overheating. The power supply, also containing the transformer driver and the control system, was fully enclosed in a separate unit. In the operating conditions, with a helium flow of 2 slpm, plasma was developed inside of the source and was propagated outside as a plume following the helium channel. The presence of the dielectric around the central electrode reduced the current transfer through the plasma to the substrate, with both thermal and electric measurements being carried out in the prototyping process to ensure that the plasma plume and the treated substrate remain at room temperature.

### 2.2. In Vitro Biological Assessment

#### 2.2.1. Cell Culture and CAP Treatment Conditions

For this study, two different cell lines, namely human gingival fibroblasts HGF-1 (CRL-2014™) and RAW 264.7 (TIB-71™) monocyte/macrophage-like cells, were purchased from American Type Culture Collection (ATCC, Manassas, VA, USA) and grown in Dulbecco’s Minimal Essential Medium (DMEM, Sigma-Aldrich Co., St. Louis, MO, USA) supplemented with 10% foetal bovine serum (FBS, Life Technologies Corporation, Grand Island, NY, USA) and 1% (*v*/*v*) penicillin (10,000 units mL^−1^)/streptomycin (10 mg mL^−1^) (Sigma-Aldrich Co., St. Louis, MO, USA). The cell cultures were maintained at 37 °C in a humidified atmosphere with 5% CO_2_, and once they reached a confluency of approximatively 80%, the cells were seeded in triplicates in 96-well tissue culture plates (TCPS) at an initial cell density of 1.5 × 10^4^ cells/cm^2^ for the gingival fibroblasts and at varying initial cell densities, depending on the biological assessment, for the RAW 264.7 cells. Thus, in order to evaluate the viability, proliferation and morphological characteristics, the macrophages were seeded at an initial cell density of 5 × 10^3^ cells/cm^2^, whereas, for the inflammatory mediators’ quantification and macrophage fusion assay, the cellular densities used were 2.5 × 10^4^ cells/cm^2^ and 2.5 × 10^3^ cells/cm^2^, respectively. Moreover, due to the fact that the periodontal disease is an inflammation-based affliction, the inflammatory activity of the RAW 264.7 cells was evaluated under both standard and pro-inflammatory (stimulation with 100 ng mL^−1^ *Escherichia coli* lipopolysaccharide (LPS, Sigma-Aldrich Co., St. Louis, MO, USA)) culture conditions.

After a time period of 24 h necessary for the cells to adhere, the HGF-1 cells and RAW 264.7 macrophages were treated directly with CAP in various experimental conditions. Thus, both cell lines were subjected to the CAP treatment once per day at different experimental time periods of 30 s, 60 s, 90 s, 120 s, 180 s and 240 s for 1 day, 3 days and 5 days. Moreover, it should be mentioned that, regardless of the experimental conditions, the distance between the cellular monolayer (bottom of the well) and the tip of the plasma source nozzle was 1.5 cm (determined in preliminary studies), and the set voltage was 2 kVpeak.

It is worth mentioning that the notations “CAP-single treatment”, “CAP-multiple (3x) treatment” and “CAP-multiple (5x) treatment” indicate how many times (days) the cells were exposed to plasma, prior to the biological evaluation. Moreover, all of the biological assays, with the exception of the fibronectin network staining, pro-inflammatory cytokine quantification and foreign body giant cells (FBGCs) formation, were conducted at 24 h after the final CAP treatment.

#### 2.2.2. Cellular Viability and Proliferation Assays

The survival of the HGF-1 fibroblasts and RAW 264.7 macrophages after CAP exposure was qualitatively investigated by using a LIVE/DEAD Viability/Cytotoxicity Kit (L-3224, Molecular Probes, Eugene, OR, USA) following the protocol described here [53]. Following staining, the cells were observed under a fluorescence microscope (Olympus IX71, Olympus, Tokyo, Japan), while an acquisition system, namely cellSense Dimension (Version 4.1), was used to capture the fluorescent images. Furthermore, the Cell Counting Kit (CCK)-8 assay (Sigma-Aldrich Co., St. Louis, MO, USA) [54] was used to quantitatively evaluate the effects of CAP treatment on the cellular proliferation rate. In the end, the optical density (OD) of the end products was determined with a microplate reader (FlexStation 3 microplate reader, Molecular Devices, San Jose, CA, USA) at 450 nm.

#### 2.2.3. Cellular Adhesion and Morphology Assessment

Considering the fact that the cell cytoskeleton is responsible for a variety of physiological events, such as stress fibres formation, cellular adhesion and morphology, in the present study, the AlexaFlour 488 phalloidin (20 µg mL^−1^, Invitrogen, Eugene, OR, USA) fluorescent staining of the actin filaments was utilised to appraise the possible effects of CAP treatment on the cytoskeleton organisation, according to a staining protocol previously described [55]. In addition, in the case of the HGF-1 cells, the indirect immunofluorescence assay [56] was also used to observe the intermediate filament vimentin—a cytoskeletal protein with a primary role in cellular adhesion and migration. In the end, the stained cells were visualised and captured with an inverted fluorescence microscope (Olympus IX71, Olympus, Tokyo, Japan) and the cellSense Dimension (Version 4.1) acquisition system, respectively.

#### 2.2.4. The Immunocytochemical Labelling of the Fibronectin Network Organised by the Gingival Fibroblasts

Since fibronectin serves as an important mediator of various cellular processes, including wound healing and inflammation, the immunocytochemical staining technique was used to reveal the fibronectin network expressed by the HGF-1 cells treated with CAP in different experimental conditions, as previously reported [57]. At the end of the immunofluorescence labelling protocol, the double-labelled cells were visualised and captured with an inverted fluorescent microscope (Olympus IX71, Olympus, Tokyo, Japan) equipped with an acquisition system (cellSense Dimension, Version 4.1).

#### 2.2.5. The Quantification of the Released Pro-Inflammatory Cytokines

The effects of CAP treatment on the pro-inflammatory mediators’ production were evaluated by measuring the concentrations of IL-6 and IL-1β secreted into the culture medium by the RAW 264.7 cells through the use of sandwich enzyme-linked immunosorbent assays (ELISA) kits, in accordance with the instructions provided by the manufacturer (R&D Systems, Minneapolis, MN, USA). The resulting ODs were measured using a microplate reader (FlexStation 3 microplate reader, Molecular Devices, San Jose, CA, USA), and the final cytokine concentrations (expressed in pg mL^−1^) were derived for each cytokine by means of its corresponding standard curve.

#### 2.2.6. Macrophage Fusion Assay

In order to study the potential of CAP treatment to mitigate macrophages to fuse and form FBGCs, an indirect immunofluorescent protocol was conducted at 7 days post-seeding. Thus, at the end of this culture period, the cells were subjected to a protocol described in another study [58] and visualised under an inverted fluorescence microscope (Olympus IX71, Olympus, Tokyo, Japan). The representative images were captured with the cellSense Dimension acquisition system (Version 4.1). Moreover, in addition to the qualitative analysis, the level of FBGCs formation was also quantified in terms of “multinuclear index”, meaning the proportion of nuclei in multinuclear cells with a minimum of 3 nuclei relative to the total number of nuclei identified within the same microscopic field.

### 2.3. Statistical Analysis

The statistical analysis was performed by means of GraphPad Prism software (Version 6, GraphPad, San Diego, CA, USA) using two-way ANOVA with Tukey’s multiple comparison tests. The values were expressed as means ± standard deviation (SD), and the *p* differences lower than 0.05 were considered as being statistically significant.

## 3. Results

### 3.1. Results Reading the CAP Source

Electrical and spectroscopical measurements have been carried out to verify the properties of the source and of the generated plasma. An upper bound on the power has been defined measuring the voltage and current upstream the transformer driver, obtaining 181 ± 1 mW, which corresponds to 9.1 ± 0.1 µJ per pulse. This value includes both the power dissipated on the plasma and the resistive losses on the different elements of the driving circuit; this upper bound, however, confirms that the source is operating in a low-power regime.

**Figure 2:**
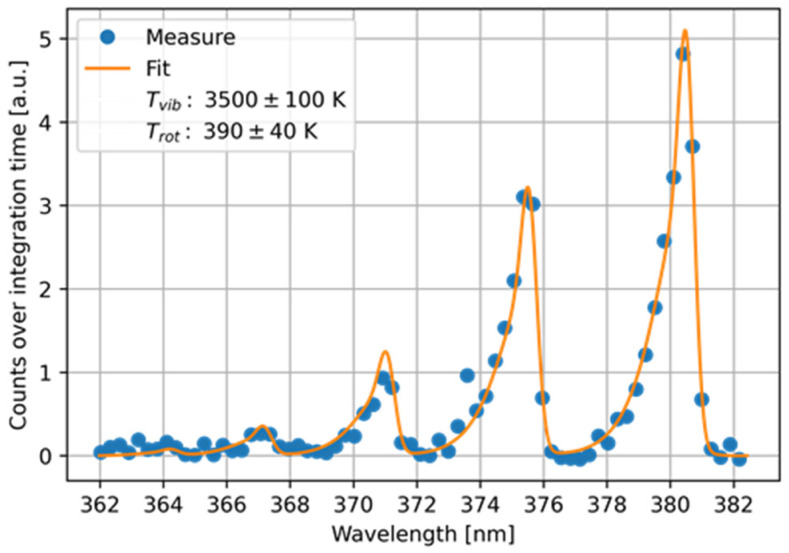
The interpolation of the nitrogen second positive system.

A preliminary characterisation of the plasma can be carried out using optical emission spectroscopy. Therefore, with the help of an Avantes AVASPEC spectrometer, the light emitted from the plasma was collected via an optical fibre during source operation in conditions comparable to the treatments. Of particular interest was the 362–382 nm range, where the emission from the nitrogen molecule second positive system was visible. Moreover, the interpolation of the measured data (Figure 2), performed using MassiveOES tools (August 2024 version) [59,60,61], allowed the estimation of the rotational and vibrational temperatures of the excited nitrogen molecules, a phenomenon which characterises the chemistry in the helium–air mixing layer. By choosing helium as the driving gas, due to its light mass and high ionisation energy (with respect to other noble gases), a high electron temperature is expected to be observed [62,63]. Thus, the interaction between the excited helium plume and air was reflected in a preferential excitation of nitrogen vibrational levels, which, in our case, was confirmed by the elevated nitrogen vibrational temperature measured at 3500 ± 100 K. However, when measured, the rotational temperature was slightly higher than room temperature, namely 390 ± 30 K.

### 3.2. In Vitro Biological Evaluation of Gingival Fibroblasts’ Behaviour After CAP Exposure

#### 3.2.1. HGF-1 Viability and Proliferation

**Figure 3:**
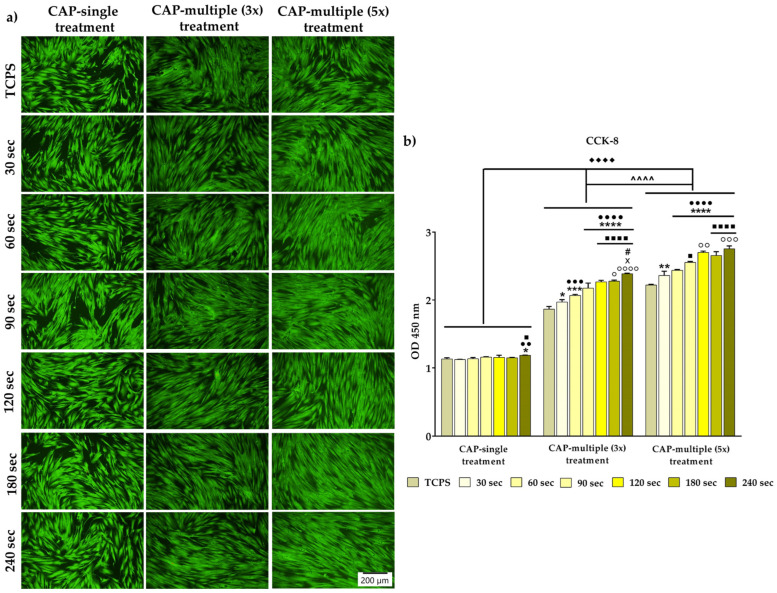
The survival/proliferation potential of the HGF-1 cells exposed to single/multiple CAP applications, as assessed by (**a**) the LIVE/DEAD assay at 24 h after the final CAP treatment (live cells: green fluorescence; dead cells: red fluorescence). The size of the scale bar is 200 µm; (**b**) the CCK-8 technique results at the same time period of 24 h after the final CAP exposure. The results are expressed as means ± SD (n = 3, **** *p* < 0.0001, *** *p* < 0.001, ** *p* < 0.01 and * *p* < 0.05 vs. TCPS; ●●●● *p* < 0.0001, ●●● *p* < 0.001 and ●● *p* < 0.01 vs. 30 s; ■■■■ *p* < 0.0001 and ■ *p* < 0.05 vs. 60 s; ○○○○ *p* < 0.0001, ○○○ *p* < 0.001, ○○ *p* < 0.01 and ○ *p* < 0.05 vs. 90 s; X *p* < 0.05 vs. 120 s; # *p* < 0.05 vs. 180 s). The significance level between the three groups: ♦♦♦♦ *p* < 0.0001 vs. CAP single treatment; **^^^^** *p* < 0.0001 vs. CAP multiple (3x) treatment.

The viability and proliferation potential of the HGF-1 cells exposed to CAP in different experimental conditions was investigated by combining the qualitative LIVE/DEAD assay with the numerical determination of the metabolically active viable cells by the CCK-8 assay. The fluorescence images presented in Figure 3a show a high percentage of green-stained live cells, with no visible dead cells (red fluorescence), regardless of treatment time and number of CAP exposures. Moreover, the repeated CAP application led to an increase in cell density, the phenomenon observed more pronounced when comparing the number of viable cells exposed to a single CAP treatment with the cellular population subjected to five consecutive CAP treatments. In line with the observations in fluorescence microscopy, the CCK-8 technique evinced statistically significant discrepancies in the analysed experimental conditions, with the non-treated HGF-1 cells exhibiting the lowest optical density (OD) values, whereas the highest proliferation rates (with an approx. increase of 30% vs. TCPS) were recorded by the fibroblasts treated for 240 s, regardless of the number of CAP applications (Figure 3b). Furthermore, the graphical analysis highlighted an increasing trend in the metabolically active viable cell population, dependent on the number of CAP applications (*p* < 0.0001). Therefore, the gingival fibroblasts exposed to a single CAP treatment exhibited a reduction of approx. 50% in their proliferation rate compared to that of the cells treated for 3 days and 5 days consecutively (*p* < 0.0001). In addition, the metabolic activity of the HGF-1 cells increased gradually with the treatment duration, a phenomenon observed more pronounced after the cells have been treated multiple times with CAP. Taken together, the obtained results demonstrate the ability of CAP to sustain the cellular viability and proliferation without exhibiting deleterious effects, especially at higher treatment times and multiple exposures.

#### 3.2.2. HGF-1 Cells’ Adhesion and Morphological Features

**Figure 4:**
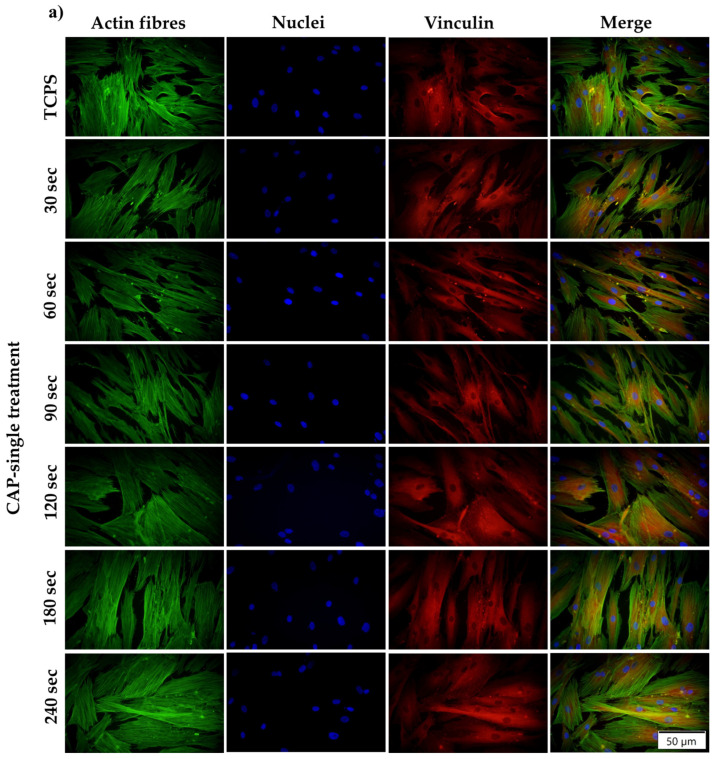

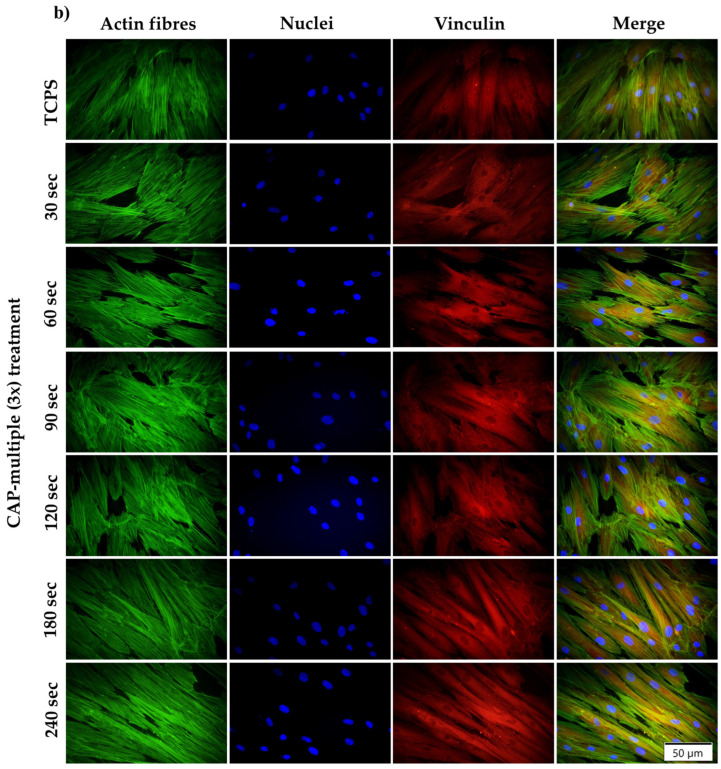
Effect of single (**a**)/multiple (**b**) CAP applications on the HGF-1 cells’ morphology, as assessed through the fluorescent labelling of the cytoskeletal proteins: actin (green fluorescence) and vinculin (red fluorescence). DAPI-labelled nuclei emit blue fluorescence. The size of the scale bar is 50 µm.

In the next experimental step, the effects of CAP application on the adhesion and morphology of the HGF-1 cells were investigated microscopically after the fluorescent labelling of two specific cytoskeleton proteins, namely vinculin and F-actin. Found abundantly in the focal contact points, vinculin is a cytoskeleton protein with a crucial role in cellular adhesion through the direct binding of actin filaments and their subsequent polymerisation and network assembly [64]. As seen in Figure 4, the microscopic examination of the fluorescence vinculin staining revealed significant differences in its distribution. In consequence, in the case of the fibroblasts exposed to a single CAP application, regardless of treatment time, the positive vinculin signals were better expressed and predominantly localised at the cells’ extremities, suggesting that a single CAP application does not affect the development of the focal contact points at the interface between the cell membrane and the plastic substrate. On the contrary, the multiple time (3 days consecutively)-treated HGF-1 cells displayed fewer focal contact points rich in vinculin at their periphery, a phenomenon also influenced by the treatment period. e.g., the number of vinculin-positive signals decreased with an increase in the treatment duration. In addition, since cell spreading is tightly regulated by the cytoskeleton organisation, the actin stress fibres were stained with AlexaFlour 488 phalloidin (Invitrogen, Eugene, OR, USA) and the fluorescent microscopy investigation revealed that the treated cells adopted different cell morphologies, depending on the experimental conditions. Thus, for the 30 s–120 s time intervals, the analysed cells adopted a typical polygonal, slightly elongated body shape, with well-expressed stress fibres and numerous cytoplasmic extensions and filopodia, whereas, for the last two time-points (180 s and 240 s), the cells displayed an increased degree of spreading, with larger bodies, for the cells treated only once and more elongated, thinner bodies for the fibroblasts treated for 3 days consecutively. To note, in both experimental conditions, a slight increase in cell density with an increase in treatment duration was observed, thus confirming the LIVE/DEAD and CCK-8 results.

#### 3.2.3. The Fibronectin Network Synthesis and Organisation

**Figure 5:**
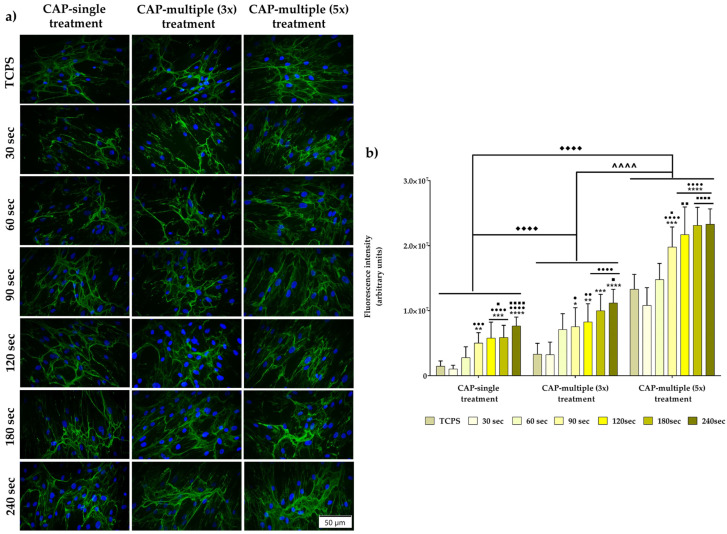
(**a**) Immunofluorescent labelling of the fibronectin network synthesised and organised by the HGF-1 cells exposed to either single or multiple CAP applications (fibronectin network—green fluorescence; nuclei—blue fluorescence). Scale bar represents 50 µm. (**b**) Fluorescence intensity measurement (n = 10, mean ± SD, **** *p* < 0.0001, *** *p* < 0.001, ** *p* < 0.01 and * *p* < 0.05 vs. TCPS; ●●●● *p* < 0.0001, ●●● *p* < 0.001, ●● *p* < 0.01 and ● *p* < 0.05 vs. 30 s; ■■■■ *p* < 0.0001, ■■ *p* < 0.01 and ■ *p* < 0.05 vs. 60 s). The significance level between the three groups: ♦♦♦♦ *p* < 0.0001 vs. CAP single treatment; **^^^^** *p* < 0.0001 vs. CAP multiple (3×) treatment.

The ECM was viewed for a very long time only as an architectural support for the connective tissue cells, when, in reality, the ECM is a complex and dynamic three-dimensional macromolecular network with tissue-specific mechanical properties involved in the modulation of several vital cellular processes such as cell proliferation, survival, migration, spreading and differentiation [65,66]. As a woven web of secreted fibrillar proteins, the ECM consists of various components, including fibronectin [67]. Thus, the evaluation of the fibronectin network organisation could provide vital information regarding the fibroblasts’ response to the CAP treatment. As seen in Figure 5a, the fibronectin network presented different distribution patterns, depending on the experimental conditions. Thus, the fibrillar fibronectin network was better expressed in the case of the HGF-1 cells treated multiple times with CAP, as opposed to a single CAP application, where, in this case, less fibrillar structures and veil-like fibronectin zones could be observed between neighbouring cells. Moreover, the quantitative determination of the fluorescence intensity (Figure 5b) corroborates the microscopical observations, revealing a statistically significant reduction in the expression of fibronectin for the cells exposed to a single CAP application. Furthermore, the evaluation of the corrected total cell fluorescence (CFTC) highlights an overall descending trend in the following order: 240 s > 180 s > 120 s > 90 s > 60 s > TCPS > 30 s, indicating a time-dependent fibronectin expression for the CAP-treated cells.

### 3.3. In Vitro Biological Evaluation of Macrophages’ Behaviour After CAP Exposure

#### 3.3.1. Macrophages’ Survival and Proliferation Rates

**Figure 6:**
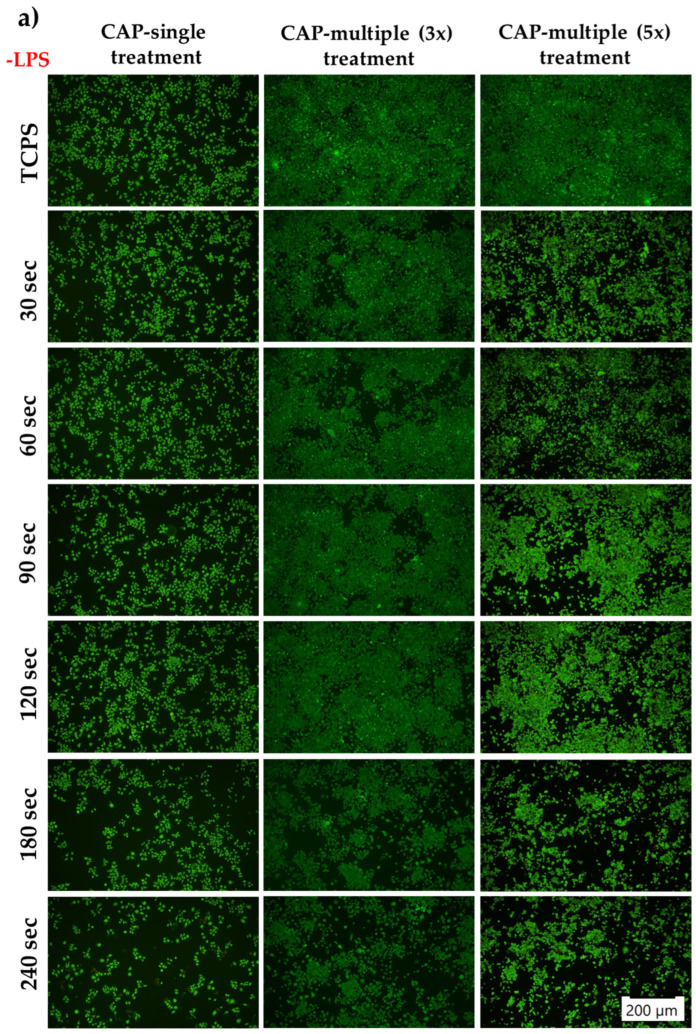

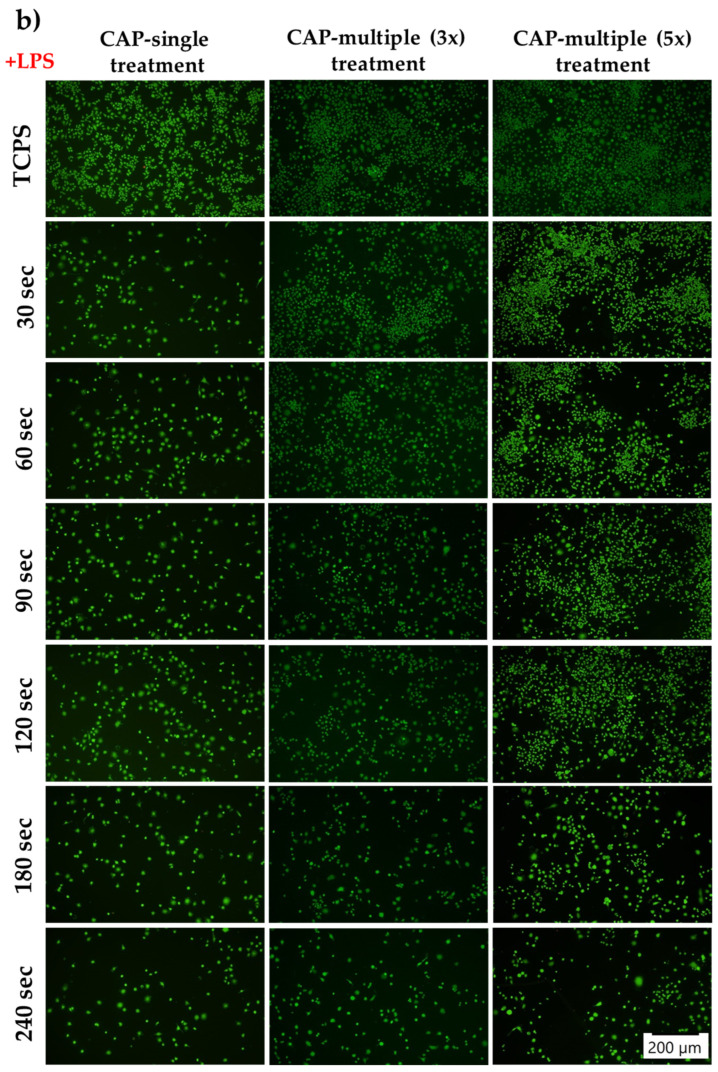
The survival potential of the RAW 264.7 cells exposed to either single or multiple CAP applications, as assessed by the LIVE/DEAD assay at 24 h after the final CAP treatment (live cells—green fluorescence; dead cells—red fluorescence) in both experimental culture conditions: (**a**) standard culture (−LPS); (**b**) macrophage activation with 100 ng mL^−1^ LPS (+LPS). The size of the scale bar is 200 µm.

The potential cytotoxic effects exerted by CAP on the macrophages’ survival rate were evaluated with the help of a LIVE/DEAD assay at 24 h post-final CAP exposure. As shown in Figure 6a, in standard culture conditions, with the exception of the cells exposed to five multiple CAP applications where the presence of dead cells (red fluorescence) was observed, the CAP-treated macrophages were able to convert calcein AM, which is non-fluorescent, to a green fluorescent calcein, indicating a high number of green-stained viable cells. Moreover, when compared to both untreated and CAP-treated cells, the fluorescence images indicated a decrease in the cellular density, proportionally with the treatment duration and number of CAP exposures, i.e., a prolonged treatment time of over 120 s and more than three CAP applications led to a numerical reduction in the cell population. Furthermore, after LPS stimulation (Figure 6b), the overall number of viable cells was found to have decreased significantly. Additionally, it is important to underline that the reduction trend in cell density dependent on treatment duration and the number of exposures was also observed for the LPS-activated macrophages.

**Figure 7:**
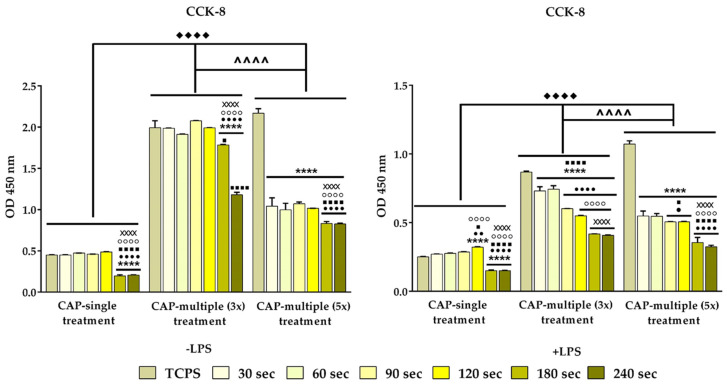
The CCK-8 assay showing the proliferative status of the RAW 264.7 macrophages exposed to either single or multiple CAP applications under both standard (−LPS) and pro-inflammatory (+LPS) conditions (n = 3, mean ± SD, **** *p* < 0.0001 vs. TCPS; ●●●● *p* < 0.0001, ●● *p* < 0.01 and ● *p* < 0.05 vs. 30 s; ■■■■ *p* < 0.0001 and ■ *p* < 0.05 vs. 60 s; ○○○○ *p* < 0.0001 vs. 90 s; XXXX *p* < 0.0001 vs. 120 s). The significance level between the three groups: ♦♦♦♦ *p* < 0.0001 vs. CAP single treatment; **^^^^** *p* < 0.0001 vs. CAP multiple (3x) treatment.

For a more in-depth evaluation of the possible deleterious effects exhibited by the plasma jet, the metabolic activity of the CAP-exposed macrophages was investigated in the same experimental conditions using the CCK-8 assay. The graphical analysis presented in Figure 7 shows a statistically significant decrease in the OD values recorded by the RAW 264.7 cells treated with plasma for 180 s and 240 s in comparison to both untreated and the rest of the CAP-treated cells (*p <* 0.0001), a trend observed regardless of the experimental conditions (absence/presence of LPS and number of CAP exposures). Additionally, in comparison to the multiple (3×) CAP treatment condition and the untreated macrophages, the cells treated 5 days consecutively with CAP exhibited a reduction of approx. 50% in their proliferation rate (*p <* 0.0001), regardless of treatment time. Furthermore, in the case of LPS stimulation, a two-fold decrease in the number of viable metabolically active cells was noticed, irrespective of the treatment time and number of CAP exposures. Moreover, it should be noted that, in the standard conditions, the highest OD values were recorded by the macrophages treated for 90 s and 120 s, whereas, in the presence of the bacterial endotoxin, the highest proliferation potential was noticed for the cells subjected to treatment with LPS for 30 s and 60 s.

#### 3.3.2. Macrophages’ Morphological Characterisation

**Figure 8:**
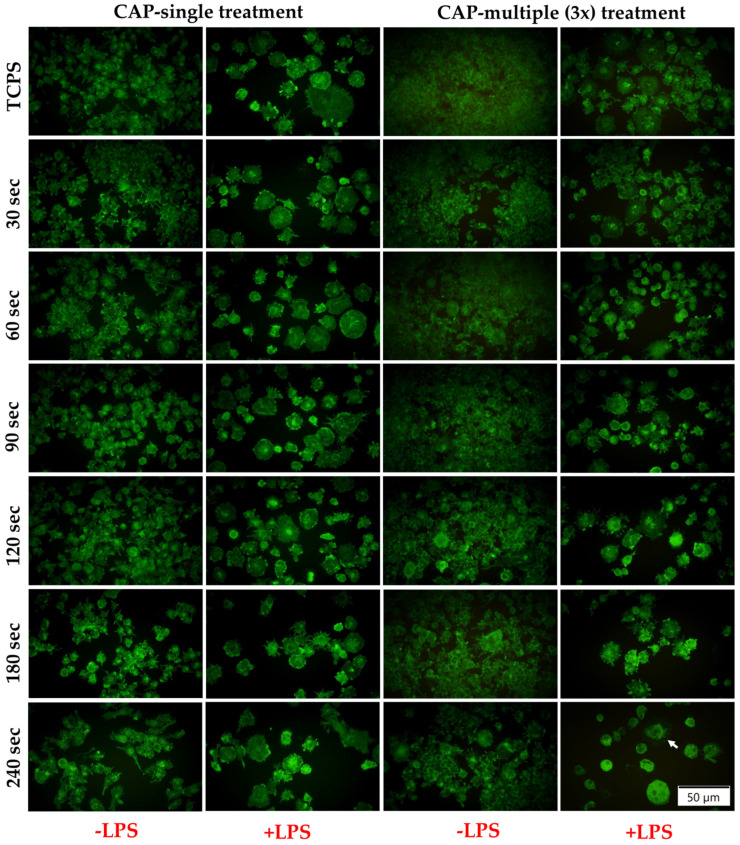
The morphological features exhibited by the RAW 264.7 macrophages after CAP treatment under standard (−LPS) and pro-inflammatory (+LPS) culture conditions (green fluorescence—actin cytoskeleton). The size of the scale bar is 50 µm.

Considering the fact that diverse microenvironmental stimuli can coerce macrophages into a phenotypic switch towards either a pro- or anti-inflammatory state characterised by a different morphology and implicit function [68,69], the macrophages’ cytoskeleton organisation was microscopically examined after labelling the actin filaments with Alexa Fluor 488 phalloidin. In consequence, in the absence of the pro-inflammatory stimulus, the microscopic evaluation (Figure 8) showed that, in comparison to the smaller, rounder, smooth-edged non-treated cells, the CAP-treated macrophages displayed a heterogenous population with slightly altered morphologies, i.e., elongated or enlarged bodies with irregular contours and numerous and more pronounced filopodial extensions. Furthermore, the fluorescence images also confirm the significant changes that the CAP-treated macrophages underwent upon the simultaneous action of LPS and CAP treatments. Thus, when stimulated, the RAW 264.7 macrophages exhibited a noticeable enhancement in their spreading area and in the number of nuclei and filopodia extensions. Additionally, the fluorescent labelling of F-actin also revealed the presence of numerous podosomes (actin structures displayed into dot-like formations) assembled in different patterns, i.e., scattered podosomes concentrated towards the extremities of the cell or, in some cases, throughout the cell’s body or large peripheral podosome clusters and ring-like super-structures. Different from the classical focal adhesion points, podosomes are cytoskeleton structures specific to macrophages, which form in the early stages of cell adhesion and consist of F-actin fibres displayed as dot-like formations on the plasma membrane extensions [70,71]. Moreover, in terms of cells’ distribution, the microscopic observations are in line with the CCK-8 test results, highlighting a significant decrease in density for the LPS-stimulated cells subjected to multiple CAP treatments but also for the macrophages treated for 180 s and 240 s, regardless of the experimental conditions and LPS presence or absence. It is worth mentioning that the RAW 264.7 cells subjected to multiple CAP applications for 240 s presented what appears to be a damaging effect in their actin cytoskeleton organisation (white arrow).

#### 3.3.3. Pro-Inflammatory Cytokine Release

**Figure 9:**
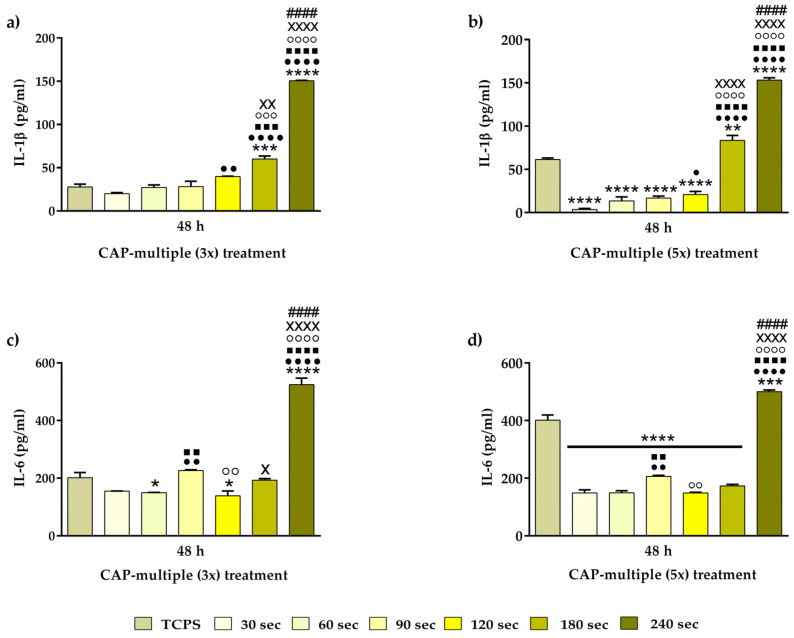
Quantification of the pro-inflammatory cytokines’ extracellular secretion by the LPS-stimulated RAW 264.7 macrophages (100 ng/mL LPS) subjected to CAP-exposure. ELISA measurement of (**a**) IL-1β—CAP multiple (3x) treatment (**** *p* < 0.0001 and *** *p* < 0.001 vs. TCPS; •••• *p* < 0.0001 and •• *p* < 0.01 vs. 30 s; ■■■■ *p* < 0.0001 and ■■■ *p* < 0.001 vs. 60 s; ○○○○ *p* < 0.0001 and ○○○ *p* < 0.001 vs.90 s; XXXX *p* < 0.0001 and XX *p* < 0.01 vs. 120 s; #### *p* < 0.0001 vs. 180 s). (**b**) IL-1β—CAP multiple (5x) treatment (**** *p* < 0.0001 and ** *p* < 0.01 vs. TCPS; •••• *p* < 0.0001 and • *p* < 0.05 vs. 30 s; ■■■■ *p* < 0.0001 vs. 60 s; ○○○○ *p* < 0.0001 vs. 90 s; XXXX *p* < 0.0001 vs. 120 s; #### *p* < 0.0001 vs. 180 s). (**c**) IL-6—CAP multiple (3x) treatment (**** *p* < 0.0001 and * *p* < 0.05 vs. TCPS; •••• *p* < 0.0001 and •• *p* < 0.01 vs. 30 s; ■■■■ *p* < 0.0001 and ■■ *p* < 0.05 vs. 60 s; ○○○○ *p* < 0.0001 and ○○ *p* < 0.01 vs. 90 s; XXXX *p* < 0.0001 and X *p* < 0.05 vs. 120 s; #### *p* < 0.0001 vs. 180 s). (**d**) IL-6—CAP multiple (5x) treatment (**** *p* < 0.0001 and *** *p* < 0.001 vs. TCPS; •••• *p* < 0.0001 and •• *p* < 0.0001 vs. 30 s; ■■■■ *p* < 0.0001 and ■■ *p* < 0.01 vs. 60 s; ○○○○ *p* < 0.0001 and ○○ *p* < 0.01 vs. 90 s; XXXX *p* < 0.0001 vs. 120 s; #### *p* < 0.0001 vs. 180 s). The results are expressed as means ± SD (n = 3).

To assess the inflammatory activity of the RAW 264.7 cells, the short-time extracellular release of the pro-inflammatory cytokines IL-1β and IL-6 was investigated under stimulation with LPS. As seen from the graphical analysis (Figure 9a,b), the expression levels of IL-1β revealed a treatment time-dependent increase, with statistically significant differences between the analysed experimental conditions. Thus, in both experimental conditions, the pro-inflammatory IL-1β cytokine levels released from the RAW 264.7 cells treated for 180 s and 240 s were notably higher (approx. two-fold and four-fold increases, respectively, for the CAP (3x) multiple treatment condition and approx. four-fold increase for the CAP (5x) multiple treatment condition) than those secreted from the non-treated and the rest of the CAP-treated macrophages (*p <* 0.0001). Moreover, a downward IL-1β expression trend, dependent on the number of CAP applications, was also observed for the cells treated at lower time intervals (30 s up to 120 s). Furthermore, it is worth mentioning that the non-treated cells recorded an approx. three-fold enhancement in the level of cytokine production as compared to the RAW 264.7 cells treated multiple times (5 days consecutively) for shorter time periods (30 s–120 s). Similar expression patterns were also observed for the pro-inflammatory cytokine IL-6, as seen in Figure 9c,d. Thus, our results indicated an increase in the IL-6 levels expressed by the cells treated for 240 s in both CAP experimental conditions. However, in contrast to the other pro-inflammatory cytokine, no significant differences were remarked in the amounts of IL-6 accumulated in the analysed culture media collected from the RAW 264.7 cells treated for shorter periods, except for the macrophages exposed to 90 s of CAP application, where increased levels of IL-6 were recorded. In addition, with the exception of the cells treated for 240 s, the non-treated macrophages secreted higher amounts of IL-6 (*p* < 0.0001) than the CAP-treated cells. Altogether, our results could suggest that, at proper operating conditions, e.g., shorter treatment times and three consecutive exposures, CAP could drive macrophage polarisation, directing the cells towards an attenuated inflammatory activity.

#### 3.3.4. Macrophage Fusion Assessment and FBGCs Formation

**Figure 10:**
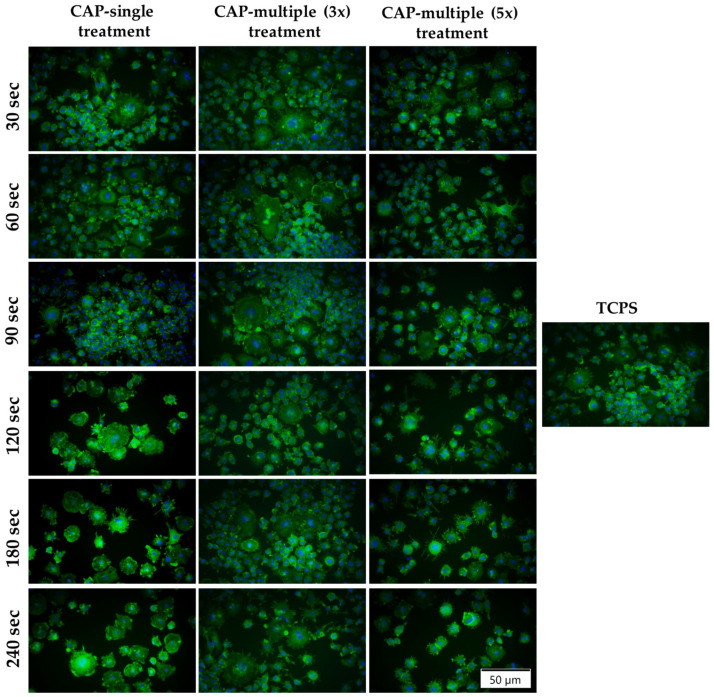
Fluorescent images of the multinucleated FBGCs generated through the RAW 264.7 macrophage fusion process after CAP treatment under stimulation with LPS (green fluorescence—actin cytoskeleton; blue fluorescence—nuclei). The size of the scale bar is 50 µm.

Since FBGCs are present only in pathological conditions and their formation is regarded in the specialised literature as a hallmark of chronic inflammation [72], in order to obtain further insight into how the CAP treatment affects the inflammatory activity of the RAW 264.7 macrophages, the capacity of CAP application to induce the formation of multinucleated giant cells was evaluated via the indirect immunofluorescence assay. After 7 days of culture, the fluorescent labelling of the actin cytoskeleton highlighted the morphological alterations that the CAP-treated RAW 264.7 macrophages underwent (Figure 10). Thus, the microscopic examination revealed a cellular population with larger bodies and multiple nuclei, a trend observed more pronounced in the case of the cells treated 1 day and 5 days for 180 s and 240 s. Moreover, even though the captured fluorescence images indicated the presence of larger multinucleated cells in the CAP (3x) multiple treatment condition, the multinuclear index as a measure of the extent of macrophage fusion revealed an overall reduction of approx. 50% when compared to the overall percentage of FBGCs generated after five multiple CAP applications. Furthermore, in terms of time-dependent FBGCs formation, the obtained values for all three analysed CAP treatment conditions increased in the following order: 30 s < 120 s < 60 s < 90 s < 180 s < 240 s (Figure S1). In addition, the value of the cells stimulated with LPS but not treated with CAP (2.1%) was lower than those of the CAP-treated macrophages, regardless of the experimental conditions.

## 4. Discussion

Due to its high efficiency, low temperature, rich reactive chemical species and low costs, CAP has rapidly gained recognition as a new and promising technology [73], with numerous uses in various areas, including the medical field, where personalised plasma-based treatments have been successfully applied in dermatology, oncology, tissue regeneration, electrosurgery and sterilisation [74]. In the field of dentistry, the starting point for CAP happened over 18 years ago, when Stoffel et al. [75] demonstrated CAP’s effectiveness in eradicating one of the most important bacteria associated with dental caries, namely the Gram-positive *Streptococcus mutans*. Since then, plasma technology has gained more and more attention as an alternative therapy for different oral health issues, such as tooth decay, candidiasis, periodontal disease, endodontic condition and even oral cancer [73].

Affecting up to 90% of the global population, in different forms and degrees of severity and complexity, periodontal disease is one of the most prevailing chronic inflammatory conditions in humans [1]. As mentioned above, one of the most common forms of periodontal disease is gingivitis, a pathological state in which various pathogens form biofilms on different parts of the buccal cavity (e.g., gingival surfaces and the tooth), triggering an unbalanced inflammatory response in the host [76], which leads to tissue damage.

According to the specialised literature, the antimicrobial efficacy of CAP is very well documented and acknowledged, with numerous papers describing the direct antimicrobial effect of CAP against various oral pathogens, such as *Porphyromonas gingivalis*, *Streptococcus mutans*, *Candida albicans*, *Prevotella intermedia* or *Fusobacterium nucleatum* [77,78,79]. However, the number of studies reporting on the regulatory effects exhibited by CAP on the oral cells is limited [80,81], despite the implication that the gingival fibroblasts and the macrophages play in gingivitis and the enormous potential that CAP may have as an adjuvant treatment strategy. With this in mind, the current study investigates the effects of a homemade plasma source on the in vitro biological behaviour of HGF-1 cells and RAW 264.7 macrophages under different experimental conditions. The obtained results revealed that CAP treatment modulates the cells’ activity in a time- and number of exposures-dependent manner, leading to an enhanced wound healing and an attenuated inflammatory state. The present observations may open the way for further in-depth studies that could cement the feasibility of CAP as a new adjuvant treatment strategy for gingivitis.

In a first step, the outcome of CAP treatment on the cellular behaviour of the HGF-1 cells was assessed in terms of cellular viability and proliferation, morphological features and fibronectin expression. Gingival fibroblasts are essential components of the periodontium and are responsible for the maintenance of the connective tissue structure and integrity [82] through the production, deposition and remodelling of the ECM [83]. Moreover, they are also involved in other biological processes such as wound healing and inflammation [84].

The LIVE/DEAD assay revealed that, regardless of treatment time and number of exposures, the cell culture maintained an increased percentage of living cells, without the noticeable presence of dead cells (red fluorescence) but with differences in the cell population number after repeated CAP applications; namely, an increase was observed for the cells exposed to multiple CAP treatments as opposed to a single CAP application. Moreover, the CCK-8 test confirmed the LIVE/DEAD results, underlying the differences in the proliferation rates of the multiple-treated cells as compared to a single CAP exposure, where the fibroblasts presented a two-fold reduction in the cell population. Similar results were also reported by Han et al. [85]. In their study, the application of a plasma jet for up to 300 s had no cytotoxic effects on the primary HGF cells’ survival and proliferation. However, data found in the literature regarding CAP toxicity is quite contradictory, with different studies reporting different exposure times as having damaging effects on the treated cells’ survival rate. For instance, Kang et al. [86] demonstrated that the CAP treatment exhibited cytotoxic effects on the viability of fibroblasts after 60 s of treatment, while Maisch et al. [87] reported a significant decrease in the fibroblasts’ survival rate after only 300 s of treatment. Considering that the toxicity of plasma is grounded in the biochemistry of the reactive oxygen and nitrogen species produced during its application, and that the amounts of said reactive species may vary widely in the culture medium of different types of cells, conflicting results could be observed due to the contrasting susceptibility of the cell type to different concentrations of reactive species (the cells’ activity is regulated in an intensity- and time-dependent manner) [88]. Thus, due to the different sensitivities of the used cell line to the created reactive species, the power of the plasma source, the exposure time and number of exposures, the comparison between the reported cell damage thresholds may not always be possible.

Moreover, the microscopic examination of the gingival fibroblasts’ cytoskeleton organisation after CAP treatment showed slight alterations in the actin fibres arrangements, with the highest treatment time-points (180 s and 240 s) leading to enlarged cellular bodies for the cells treated only once and to more elongated bodies for the cells treated multiple times. In addition, to further observe the fibroblasts’ response to CAP, the fibronectin network organisation was evaluated. Less studied than other major components of the ECM, e.g., collagen, fibronectin is an abundant matrix protein playing important roles in cellular adhesion, actin cytoskeleton organisation and wound healing [89]. From a structural point of view, fibronectin is a dimeric adhesive glycoprotein composed of repetitive units of amino acids grouped into domains, an arrangement that allows the protein to interact with different types of cells by means of both non-integrin and integrin receptors [90]. Moreover, even though fibronectin is encoded by a single gene, due to the pre-mRNA alternative splicing process, it exists under different multiple forms (20 isoforms), which are involved in adhesion-regulated survival signalling and cellular proliferation and differentiation [66]. The arginine-glycine-aspartic acid (RGD) cell binding site enables fibronectin to connect with α5β1 integrins, forming a prototypic integrin–ligand complex. This complex mediates the contractility of cells and integrin clustering through the reorganisation of the actin cytoskeleton structure [90]. This, in turn, leads to conformational alterations in the fibronectin dimers binding to the α5β1 integrins, revealing hidden sites within the fibronectin molecule, which drives the multimerisation process through self-interactions [67]. In the end, fibronectin is assembled, initially into fine cell-associated fibrils, which, through its continuous accumulation, will end up being organised into a condensed, stable extracellular fibrillar network that extends between neighbouring cells [57]. In our study, the immunocytochemical staining assessment revealed different distribution patterns, with a network of fibronectin better expressed and organised, in the case of the gingival fibroblasts exposed to multiple CAP treatments. Furthermore, the evaluation of the corrected total cell fluorescence (CFTC) showed a time-dependent fibronectin expression, with an overall descending trend from the highest time-point to the lowest.

In the second part of the study, the cellular behaviour of the RAW 264.7 macrophages was investigated in terms of survival/proliferation, morphological characterisation and inflammatory response. Since gingivitis has an inflammatory component, the host’s innate immune system has a decisive role in the initiation of the immune response towards the microbial pathogens [91]; therefore, an impaired innate immunity signalling can increase the severity of the condition [92]. On the other hand, tissue injury and a more severe inflammatory state can occur due to an over-activated immune response [91], the reason why the modulation of the main effector cells of the immune system, namely macrophages, towards an anti-inflammatory phenotype is most desirable.

In contrast to the HGF-1 cells, the CAP treatment exhibited deleterious effects on the RAW 247.7 cells’ survival and proliferation rates, which were proportional with the treatment duration and number of CAP applications, regardless of the experimental conditions (absence or presence of LPS stimulation). Moreover, by pre-treating the cells with bacterial endotoxin LPS, an overall reduction in the number of metabolically active viable cells was observed, a phenomenon that could be ascribed to the inhibitory effect of the bacterial pro-inflammatory agent (LPS) on the cellular viability/proliferation processes, rather than that of the CAP treatment. Overall, the cytotoxicity study indicated that, in the case of the RAW 264.7 macrophages, the most beneficial effects were exerted after exposing the cells to no more than three consecutive CAP applications and only up to 120 s. Our findings are in accordance with previous studies, where time-dependent deleterious effects on the macrophages’ viability/proliferation rates were observed. For example, in a paper by Hirwasawa et al. [93], LPS-stimulated THP-1 cells were exposed to CAP, and the evaluation of the possible toxic effects against these macrophages showed the absence of deleterious effects following a period of less than 70 s of irradiation, whereas, after longer treatment times, namely 150 s, the THP-1 cells exhibited a reduction in their survival/proliferation rate. Moreover, Crestale et al. [80] evinced a time-dependent reduction in the cells’ viability rate, proportional with the increasing concentrations of reactive species generated with longer treatment times. To further confirm the observations from the cytotoxicity study, the cytoskeleton organisation of the RAW 264.7 cells was microscopically examined after the actin filaments have been stained with a fluorophore and the fluorescence images revealed that, in the LPS absence, the cell culture presented macrophages with mixed morphological features, whereas the LPS pre-stimulated cells displayed a morphological behaviour associated with the pro-inflammatory M1 phenotype [94]. In addition, the microscopic observation confirmed the cytotoxicity results, outlining the deleterious effects of a prolonged CAP exposure, visible through the significant decrease in cell density, regardless of the experimental conditions and LPS presence or absence.

Finally, to evaluate the CAP’s effect on the inflammatory activity of the RAW 264.7 macrophages, the pro-inflammatory cytokine secretion profile and FBGCs formation were assessed. Similar to other biological barriers (e.g., respiratory and gastrointestinal tracts), the periodontal tissue is constantly subjected to various buccal microorganisms and to a plethora of chemical and physical factors produced during respiration and mastication [95]. However, under physiological conditions, the immune acceptance and monitoring of the local microbiota is attained without eliciting a severe inflammatory reaction through the establishment of a delicate balance between the two [96]. Nevertheless, after pathogenic bacterial colonisation, the local microbiota suffers a series of alterations, which increases the overall pathogenicity of the bacterial community and disrupts tissue homeostasis [97]. Thus, under these conditions, the host’s innate immune system is activated, and several pro-inflammatory mediators such as cytokines and chemokines are released into the microenvironment, contributing to the immune system’s further activation [98]. Furthermore, the host’s acquired immune system can also be activated by pathogenic bacteria, leading to a more severe inflammatory response and a more rapid progression of the periodontal disease [99]. As the inflammatory process continues, the secreted pro-inflammatory mediators can severely affect the periodontal structures (e.g., gingiva, periodontal ligaments and alveolar bone), causing permanent and irreversible bone damage and the loss of periodontal attachment [98].

Cytokines are highly important extracellular signalling proteins released from a variety of cells (e.g., lymphocytes, macrophages, natural killer (NK) cells, mast cells and stromal cells), with key roles in immunity, inflammation, fibrosis and wound healing [100,101]. Furthermore, they also mediate several important cellular processes, such as gene expression, cell migration, proliferation and differentiation [99]. From data reported in the literature, it became clear that the inflammatory process of gingivitis is accompanied by the presence of high concentrations of several pro-inflammatory mediators (e.g., IL-1β, IL-6, TNF-α and interferon (IFN)-γ) [98] and that, by inhibiting said inflammatory mediators (e.g., TNF-α and IL-1), an attenuated inflammatory response was observed [102]. Taking this into account, the short-time secretion of IL-1β and IL-6 was investigated after the cells were pre-treated with the bacterial endotoxin LPS. IL-1β is a pro-inflammatory cytokine that is induced following host–microbiota interactions and plays a role in the pathogenesis of periodontal disease, influencing the expansion and activation of both Th1 and Th2 cells [103]. Similar, IL-6 is a pro-inflammatory cytokine with a very well documented role in periodontal disease, having been demonstrated to actively participate in the initiation and the acute phase of the disease [104,105]. The obtained results indicated that the IL-6 and IL-1β concentrations exhibited a treatment time-dependent increase, with the highest cytokine levels being recorded by the RAW 264.7 cells exposed for 180 s and 240 s to CAP. Of particular interest is the fact that the secreted levels of both pro-inflammatory cytokines accumulated in the cell culture media of the macrophages treated for no longer than 90 s were lower as opposed to the positive control group, thus indicating a less severe inflammatory initial macrophage response upon CAP application. Complementary to the cytokine secretion profile, the FBGCs formation revealed that the cells treated 1 day and 5 days consecutively for 180 s and 240 s acquired a more pronounced altered morphology and a higher multinuclear index, indicating that these plasma source operating conditions may sustain the unwanted chronic inflammatory state. Taken together, the inflammatory activity assessment suggests that, at proper operating conditions, e.g., shorter treatment times (e.g., 90 s) and 3 consecutively exposures, CAP could regulate the macrophage polarisation state, directing the cells towards an attenuated inflammatory response.

## 5. Conclusions

Overall, our results showed that the multiple (3x) direct application of CAP for 90/120 s had beneficial effects on cell adhesion and proliferation, without eliciting cytotoxic effects for both gingival fibroblasts and macrophages. Since ROS generated by CAP regulate the cellular behaviour in an intensity- and time-dependent manner, we hypothesised that the above indicated CAP exposure time parameters led to the production of a low level of ROS, which aided cellular survival and proliferation. Moreover, a prolonged treatment time (over 120 s) resulted in an intense pro-inflammatory response, as evinced by the macrophages’ morphological alterations, the formation of FBGCs and the pro-inflammatory cytokine profile. Although further in vitro (on bacteria and mammalian cells) and in vivo studies are required, the present work paves the way and brings forth knowledge that, in the future, will help to ensure the safe and effective translation of CAP from research to clinic as an alternative treatment for gingivitis.

## Data Availability

Data are contained within the article.

## Supplementary Materials

The following supporting information can be downloaded at https://www.mdpi.com/article/10.3390/cells13231970/s1: Figure S1: The values of the “multinuclear index”, as determined by examining 10–14 microscopic fields for each sample.
