# Supplementary material for: The Potential of a Novel Cold Atmospheric Plasma Jet as a Feasible Therapeutic Strategy for Gingivitis—A Cell-Based Study
Source: Cells. 2024 Nov 28;13(23):1970. doi: 10.3390/cells13231970 (PMC11640168; doi:10.3390/cells13231970)
Supplement: Supplementary file 1 [file cells-13-01970-s001.zip › cells-3329949-supplementary.pdf]

Supplementary Material

The Potential of a Novel Cold Atmospheric Plasma Jet as a Feasible Therapeutic Strategy for Gingivitis —A Cell-Based Study

Andreea-Mariana Negrescu[1,2†], Leonardo Zampieri[3†], Emilio Martines[3], Anisoara Cimpean[1*]

Sample 100 ng mL^{-1} LPS	Total number of nuclei	Number of nuclei in FBGCs	Multinuclear index (%)
Control			
TCPS	190	4	2.1%

Sample 100 ng mL^{-1} LPS	Total number of nuclei	Number of nuclei in FBGCs	Multinuclear index (%)
CAP-single treatment			
30 sec	125	3	2.4%
60 sec	124	6	4.8%
90 sec	186	9	4.8%
120 sec	68	3	4.4%
180 sec	30	6	5%
240 sec	39	5	7.6%

Sample 100 ng mL^{-1} LPS	Total number of nuclei	Number of nuclei in FBGCs	Multinuclear index (%)
CAP-multiple (3x) treatment			
30 sec	145	5	3.4%
60 sec	182	8	4.3%
90 sec	116	5	4.3%
120 sec	95	4	4.2%
180 sec	119	7	5.2%
240 sec	98	6	6.1%

Sample 100 ng mL^{-1} LPS	Total number of nuclei	Number of nuclei in FBGCs	Multinuclear index (%)
CAP-multiple (5x) treatment			
30 sec	89	6	6.7%
60 sec	71	6	8.4%
90 sec	73	7	9.5%
120 sec	58	5	8.6%
180 sec	64	5	7.8%
240 sec	43	5	11.6%

Figure S1. The values of the "multinuclear index" as determined by examining 10–14 microscopic fields for each sample.
